# Medical Doctors Approaches and Understanding of Health Literacy: A Systematic Literature Review

**DOI:** 10.7759/cureus.51448

**Published:** 2024-01-01

**Authors:** Eleni Louizou, Nikolaos Panagiotou, Eleni Dafli, Emmanouil Smyrnakis, Panagiotis D Bamidis

**Affiliations:** 1 School of Medicine, Faculty of Health Sciences, Aristotle University of Thessaloniki, Thessaloniki, GRC; 2 School of Journalism & Mass Communications, Aristotle University of Thessaloniki, Thessaloniki, GRC; 3 Primary Health Care, General Practice and Health Services Research, Faculty of Health Sciences, Aristotle University of Thessaloniki, Thessaloniki, GRC

**Keywords:** medical doctors, health literacy, systematic literature review, qualitative studies and mixed-methods studies, medical education, physicians

## Abstract

A physician's role is critical in fostering patient health literacy (HL) and influencing various aspects, including patient-physician communication and treatment effectiveness. The purpose of this systematic literature review is to analyze physicians' perspectives, comprehension, and management of HL. The focus of this review is on physicians' views, opinions, experiences, and strategies related to HL.

We conducted comprehensive searches across seven databases, including PubMed, Scopus, ProQuest, Science Direct, Web of Science, The Cochrane Library, and Google Scholar. Original research articles published between January 1, 2009, and July 31, 2020, were considered for inclusion. This literature review incorporates qualitative studies and mixed-methods studies, with a focus on extracting qualitative data. Among the 22 articles included in our review, we employed the method of inductive thematic analysis for data analysis. A detailed description of the review methodology can be found in a previously published protocol available through PROSPERO (CRD42020212599).

The themes that emerged from the thematic analysis include: (a) physicians' perception and management of HL; and (b) barriers. The results of the systematic review reveal that healthcare professionals exhibit varying perceptions of patients' HL levels and ascribe different meanings to it. However, none of them employ a specific measuring tool. While there appears to be no uniform approach to managing patients with low HL, some prioritize certain communication strategies, such as repetition, simplified language, and providing written instructions, among others. Most physicians cited multiple barriers that impede the development of patients' HL, including dysfunctions within the healthcare system, staff shortages, managing a large number of patients, limited time, work-related stress, cultural and socio-economic barriers, medical jargon, and language barriers.

Considering the pivotal role of physicians in fostering patient HL, it is crucial to enhance medical education in addressing and managing HL, both within academic curricula and through continuing education seminars. Furthermore, there is a pressing need to improve healthcare professionals' working conditions, ensuring that each physician can allocate the necessary time to each patient based on their individual needs, without being hindered by stress-inducing work environments.

## Introduction and background

Health literacy (HL) is vital in the maintenance and improvement of individual health and well-being and plays a crucial role to prevent and better manage health problems. While there has been attention to the implications of HL in individual and public health, little attention has been paid to how medical doctors perceive, understand and manage HL. With this knowledge, we can better address HL strengthening the role/contribution of medical doctors in developing the HL of their patients.

HL is a term developed in the United States in the 1970s and has been evolving ever since, with significant and growing importance to public health and healthcare [1]. It entails people's knowledge, motivation and competences to access, understand, appraise, and apply health information in order to make judgments and take decisions in everyday life concerning healthcare, disease prevention and health promotion to maintain or improve quality of life during the life course [2]. HL is associated with self-management skills and improved health outcomes in a range of chronic diseases [3-8]. It is also associated with a reduction in the likelihood of having a comorbid condition [9]. Individuals with low HL had a higher probability of emergency department revisits and had greater healthcare utilization and expenditures spending more on prescriptions while HL was essential to improving medication adherence [10-12]. Αlso have been demonstrated the efficacy of HL interventions especially among more vulnerable patient groups [13].

Over the past decade, several systematic reviews have documented patient’s HL [14-17]. One systematic review has investigated HL among people with intellectual disabilities [18]. Another systematic review focused on HL among students of health professions and their clinical educators [19]. One systematic review that investigated multifaceted interventions aiming to improve health of people with limited HL also identified two studies focusing on health professionals in general as targets of a relevant intervention [20]. In another systematic review that investigated enabling and hindering factors influencing adherence to asthma treatment among adolescents, the role of caregivers and healthcare providers was explored in terms of supporting adolescents to effectively manage and live with asthma [21]. Finally, another review focused on the perspectives on HL of both healthcare providers in general (including nursing students) and patients and included studies that employed both qualitative and/or quantitative descriptions of HL [22]. To the authors’ best knowledge, this is the first systematic literature review to specifically investigate medical professionals’ ability to understand and manage HL issues.

The aim of the systematic literature review is to explore physicians' perception and understanding of HL. In this context, this systematic review will attempt to answer the following research question: How do physicians manage HL issues? This systematic review will focus on views, opinions, experiences and management, in relation to HL in physicians. The review question is best answered through a qualitative study. In addition, mixed-methods studies were also included provided they contain relevant qualitative data.

## Review

Methods

The ENTREQ statement was used as a tool to enhance transparency, in reference to the reporting guidelines for the synthesis of qualitative research [23]. The methodology of this review has been described in detail in a previously published protocol through PROSPERO (CRD42020212599).

Search Strategy

The search was performed in the following databases: PubMed, Scopus, ProQuest, Science Direct, Web of Science, the Cochrane Library and Google Scholar (the 100 most recent results were taken into account). The search was updated before the final analyses and new entries were considered for inclusion.

Admission Criteria

Articles with a publication date falling between January 1, 2009 and December 31, 2019 were included. Searches were repeated just before the data collection stage, from May 23, 2020 to July 31, 2020, to include studies published in the intervening time period. "Grey literature" was excluded from the systematic literature review. Original research articles only were included.

Sample

The research focused exclusively on the perspective of medical professionals including general and specialist physicians (i.e., general practitioners, obstetricians, surgeons etc.). Other health professional were excluded (i.e., nurses, midwives, health workers, pharmacists, dentists etc.). In addition, psychiatrists were excluded from the selected sample as they fall under the specific category of mental HL. Medical students were also excluded but physicians in residency (i.e., physicians in training for a given specialty) were included.

Data Extraction

After we have identified all study records using the search strategy, duplicate entries were removed from the dataset. The titles and abstracts of the remaining records were thoroughly screened to assess their relevance, in accordance with the inclusion and exclusion criteria. Studies that passed this initial screening were then included for a comprehensive review of their full texts. The full texts of the pertinent studies were thoroughly examined to ascertain whether they met the specified inclusion criteria.

The risk of bias reduced with the involvement of two reviewers independently assessing study eligibility and quality. Any potential discrepancies between the selected manuscripts were resolved through discussions between the two authors. In instances where a consensus agreement could not be reached, a third author was consulted to make the final decision.

Papers that were included in the final review were uploaded into NVivo 12 Plus. One author extracted the data independently and another author reviewed a portion to ensure the process is being done correctly. First order constructs were extracted and analyzed to produce an interpretive synthesis of the data.

Critical Evaluation

After the selection process was completed, the resulting articles were evaluated for their methodological rigor using the critical evaluation tool called Critical Appraisal Skills Programme (CASP) [24]. A quality assessment was carried out for each article. Each of the 10 questions was scored with a scoring system (Yes: 1 point, Unsure: 0.5 points, No: 0 points) and low quality papers (scoring less than 6) were excluded.

Data Composition

The method of thematic analysis was chosen for data analysis [25]. The topics emerged directly from the data, without a predefined coding framework, using an inductive analysis, while also striving to maintain some connection with the research questions.

Results

**Figure 1:**
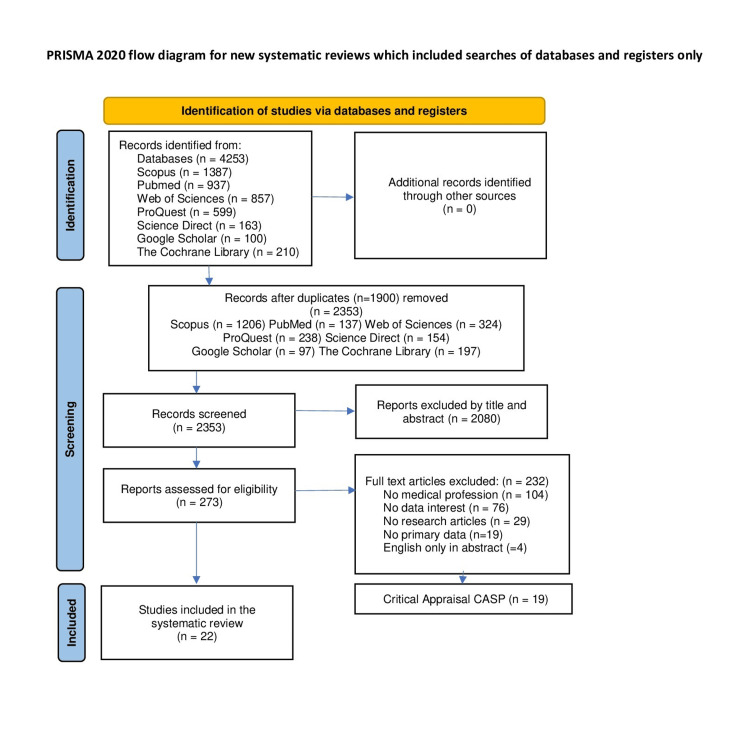
PRISMA flowchart

Figure 1 shows the flow of articles during the review process [26].

The 22 articles answering the research question of the literature review are summarized in Table 1 [27-48]. These are mainly qualitative studies using in-depth semi-structured interviews with physicians and there are four studies based on mixed methods, from which only the qualitative data that emerged were utilized.

**Table 1:** Characteristics of the included studies HL: Health literacy; CALD: Culturally and linguistically diverse; HPs: Health professionals; OHC: Online health community

Α/Α	Author	Country of origin	Μethodology	Findings about HL
1	Alzaye et al., 2019 [27]	Australia	Qualitative	This study has provided insight into the notion that GPs who deal with CALD patients with asthma face more challenges, because of HL, among other factors.
2	Atanasova et al., 2017 [28]	Slovenia	Qualitative	HPs who engage in OHCs, as moderators perceive themselves as facilitators of patients and other OHC’s users' empowering processes and outcomes, among which is the improvement of their HL.
3	Baars et al., 2017 [29]	Germany	Mixed methods	The most important determinants for non-participation in genetic counseling were experienced difficulties in patient-doctor communication, limited HL etc. Patients and HPs experience significant language and HL difficulties, which make it harder to fully access health care such as genetic counseling and testing.
4	Caiata-Zufferey & Schulz, 2012 [30]	Switzerland	Qualitative	A typology of four communicative strategies has been outlined. The adoption of these strategies is shaped by physicians’ general attitude toward Internet-informed patients, based on their conception of medical information for lay people through the Internet. This general attitude is mediated by doctors’ interpretation of the specific communicative context, that is, their appraisal of three aspects, one of which is the patient's HL among others.
5	Eraso, 2019 [31]	Argentina	Qualitative	Factors influencing prescribing practices of hormonal therapy are varied. Women’s socio-economic status and their level of HL can affect oncologists’ prescribing practices.
6	Faruqi et al., 2015 [32]	Australia	Mixed methods	HPs described three approaches to preventive care, for primary care patients with low HL, which remained largely unchanged. They demonstrated recognition of the importance of better communication and referral support for patients with low HL. However, they identified fewer patients with low HL than anticipated, highlighting the need for increased attention to HPs’ attitudes in supporting these individuals.
7	Hughson et al., 2018 [33]	Australia	Qualitative	There are significant HL and systemic issues affecting the hospital’s provision of maternity care for CALD women. Emergent themes of HL-related issues were: patient-based factors (communication and cultural barriers, access issues); provider-based factors (time constraints, interpreter issues); and enablers (cultural awareness among staff, technology).
8	Kaplan et al., 2016 [34]	USA	Qualitative	The findings underscore the importance of the physician-patient relationship as a point of intervention to address the unmet informational and psychosocial needs of Latino cancer patients. Patients and HPs tended to differ on the value of print materials. HPs tended to view them as inadequate for meeting the informational needs of their Latino patients due to the challenge of low HL. HPs mentioned low HL as a barrier.
9	Khuu et al., 2016 [35]	USA	Qualitative	In order to investigate HPs' perspectives on the HL of immigrant and refugee parents and its association with children's health, six broad themes were identified: (1) multi-dimensional components of parental HL; (2) parent characteristics and native country experiences; (3) host systems and their interactions impact on parental HL; (4) diverse aspects of help-seeking; (5) culture-based parental help-seeking; and (6) child health outcomes.
10	Lamb, 2018 [36]	USA	Qualitative	Patient–physician shared decision making is bounded/limited by “nudging” bias, power balance considerations, and consideration of patient HL alignment.
11	Lambert et al., 2014 [37]	New Zealand, Australia & Canada	Qualitative	HPs have a limited understanding of HL and of the consequences of low HL for their indigenous patients. This lack of understanding combined with the perceived barriers to improving HL limit HPs’ ability to improve their indigenous patients’ HL skills.
12	Litchfield et al., 2014 [38]	UK	Qualitative	With the aim of understanding how laboratory test results are conveyed to patients in primary care and gathering insights on how the process can be enhanced, five primary themes emerged from the data. One of these themes pertains to the clinical impact of results and how patient characteristics, such as anxiety level or HL, influence the methods through which patients receive their test results.
13	Oslislo et al., 2019 [39]	Germany	Qualitative	With the aim to explore the GPs’ view regarding motives and competences of patients self-referring to emergency departments it became evident that counselling efforts by GP were described as crucial for improving HL. Physicians main criticism was notably directed at HL and patients’ competence to assess their own symptoms.
14	Pennington et al., 2017 [40]	Australia	Qualitative	Evaluating a woman's HL level is crucial, as it signifies the role she envisions for her GP in her engagement with diabetes preventative care following a pregnancy with gestational diabetes.
15	Periyakoil et al., 2015 [41]	USA	Mixed methods	One of the biggest doctor-reported barriers to effective end-of-life conversations was patients’ limited HL.
16	Sadeghi et al., 2013 [42]	Canada	Qualitative	The findings provide an increased understanding of patients’ and HPs’ perceptions of HL as a barrier to effective communication of medical information to patients with chronic obstructive pulmonary disease as well as approaches that might improve this communication.
17	Salter et al., 2014 [43]	UK	Qualitative	Gaps in conceptualization and expectations were revealed, reiterating deficiencies in predominant models for understanding HL and methodological shortcomings for this topic. Findings on factors perceived to foster and inhibit HL and on the issue of responsibility in HL. HPs had more heterogeneous views. All participants agreed that HL most benefited from good inter-personal communication and partnership.
18	Schulman-Green et al., 2018 [44]	USA	Mixed methods	Goals of care conversations may be facilitated by enabling oncologists to conduct these conversations despite difficult circumstances and emotional reactions by activating patients and family via increased HL and by advancing palliative-informed practice environments.
19	Smith et al., 2014 [45]	Australia	Qualitative	Radiation oncologists described subjectively assessing a person’s HL level by monitoring the types of questions asked; analyzing the language used; examining non-verbal behaviour, and considering a person’s socio-economic situation. Participants reported the challenges of discussing the subtleties of cancer treatments with lower HL groups.
20	Walton et al., 2018 [46]	UK	Qualitative	Socioeconomic deprivation influences GP referral decisions and navigation of the healthcare system in multiple ways. GPs perceived particular problems due to patients with lower HL in more deprived areas, making the identification of problems possibly needing referral more complex.
21	Zafar et al., 2016 [47]	USA	Qualitative	Some primary HPs reported using a uniform approach to communicate and manage incidental findings, while others adapted their approach to the patient and the finding. Sometimes patient characteristics such as HL superseded HPs characteristics.
22	Zanini et al., 2019 [48]	Switzerland	Qualitative	Building and maintaining a partnership with individuals with spinal cord injury to prevent and treat pressure injuries is crucial, but it is not an easy task HPs. Specific communication skills can help HPs. The HPs presented themselves as tutors during rehabilitation, in that they play a key role in educating patients and building HL for autonomous decision-making.

The themes that emerged from the thematic analysis of the physicians' interviews and were considered important for the research questions posed are the following:

1. Physicians perception and management of HL

2. Barriers

The categories of topics and sub-topics of the articles are presented in Table 2.

**Table 2:** Number of studies categorised under themes and sub-themes HL: Health literacy

Τhemes	Number of studies categorised under	Sub-themes	Number of studies categorised under
Theme 1: Physicians’ perception and management of HL	16	Ways physicians perceive and understand patient HL	9
		Management of patients with low HL	13
Theme 2: Barriers	20	Systemic factors	5
		Time constraints	9
		Cultural barriers	8
		Socio-economic barriers	10
		Language	8
		Medical jargon	5

Physicians’ perception and management of HL

The way in which physicians perceive and manage their patients' HL, as well as the significance they attributed to it, are presented in this theme.

Ways physicians perceive and understand patient HL

From physicians responses in the interviews conducted in the articles, it was evident that they varied in their perception of a patient's HL level and attributed different meanings to it. In certain articles, some physicians argued that a significant component of HL was related to the patient's ability to navigate and utilize the healthcare system, where individuals with low HL encountered the most difficulties [27,35,37,41,45,46].

“But my understanding [health literacy] is people having a sort of workable knowledge of how they interact with the health system.” (Physician) [37]

 

“This general, let's say distrust or lack of like familiarity with medical practice, doctors… you don't go there until you're dying.” (Physician) [35]

 

“They may not be used to the health system they find themselves in and it may be overlooked that they lack what we would consider common knowledge.” (Physician) [41]

In certain articles, some physicians argued that a crucial aspect of HL was the patient’s ability to comprehend and manage their prescribed medications, with individuals possessing lower HL facing more significant challenges [27,31,37,45].

“Many times it has happened to us that we give them the medication and they return in three months, and they say ‘I took it once Dr.’, this means that often they don’t understand the treatment even when you write it down for them, and even when they know how to read, they don’t understand it.” (Physician) [31]

Physicians have observed that patients with low HL tend to exhibit a “don't tell me” attitude towards their doctors. They often visit the physician when their condition has progressed significantly, either asking repetitive questions or seeking clarification on matters already discussed. Additionally, they may not realize the significance of vaccination. [35,45]

In terms of patients' self-management of their health, in other studies, physicians have noted that individuals with low HL lacked the ability to effectively organize themselves [39,45]. Additionally, they exhibited a limited understanding of the available resources and treatments [41].

“Incomplete understanding of what resources/therapies that can be versus should be provided for a patient.” (Physician) [41].

Regarding the level of responsibility a healthcare professional could assume in the development of HL, a study by Lambert et al. indicated that some physicians were more inclined to view the lack of HL as a 'defect' of the patient rather than a responsibility of the physician to enhance the patient's HL [37]. On the other hand, in different instances, some physicians appeared more willing to take on the responsibility for improving the HL of their patients, as observed in other studies [27,43]. One physician appeared to indirectly acknowledge their responsibility in patient health education, stating that:

“The more I explain, the more the patient knows. The more he knows, the more competent he becomes (…). If I explain well, people are more competent. And health education is important (…).” (Physician) [39].

Management of Patients with Low HL

Regarding the management of patients with low HL, in some articles, physicians supported different approaches and founded various strategies to assist patients with low HL. Repetition or breaking down the information and providing it throughout their treatment, rather than all at once [32,34] were among the communication strategies mentioned as being followed by some physicians.

“People with low health literacy do need the repetition, they may gain 5% of the information each time, but they do gain something each time.” (Physician) [32].

In another study, it was mentioned that when physicians were managing patients with low HL, they tended to provide more written instructions. They also frequently paused during conversations to confirm the patient's understanding or spoke slowly or used simple language, and encouraged the patients to ask straightforward questions [45].

“I’m picking up on clues about whether or not they’re actually understanding what’s been said. I guess giving them the opportunity to ask plain questions and then make a judgment about how much they’ve understood based on the kinds of questions.” (Physician) [45].

Additionally, in an article exploring the strategies employed by physicians when interacting with Internet-informed patients, a perceived low level of patients' HL encouraged physicians to adopt a closed-minded stance towards Internet information, whereas a perceived high level prompted them to be more receptive [30].

In another article that aimed to identify HL issues in the provision of maternity care to culturally and linguistically diverse (CALD) women and the strategies required for healthcare professionals to collaboratively address these issues, a physician advocated for the use of technology to assist in managing the needs of these individuals [33].

“I actually do genuinely believe that technology, used properly, and in a very specific and focussed way, is probably the solution. I think the one thing that ties everything together, or could tie everything together, around the patient, would be technology.” (Physician) [33].

The approach employed by physicians in managing their patients and selecting a communication method to convey the results of medical examinations depended on patient-specific characteristics, including anxiety levels or HL [38,47].

“I think knowing your patient and the level of anxiety it’s going to cause. So if you think that by phoning or sending a letter is going to cause them anxiety you would actually phone them.” (Physician) [38].

The patient's level of HL appeared to have a significant impact on whether the physician will allow the patient's participation in the choice of treatment. Some physicians seemed to be more inclined to involve patients with high HL in the participatory decision-making process:

“High literacy patients, I think, have the opportunity to be more actively involved in treatment choice.” (Physician) [45].

Some physicians have noticed that individuals with high HL tend to have greater autonomy in making decisions about their health [45]. Having high HL might provide them with more confidence than they required, which did not necessarily lead them to make the right decisions for their health outcomes [36].

“In fact, sometimes people who are very highly educated go out there and make up their own mind what they want to do, and it was a pretty dumb decision.” (Physician) [36].

Trust in the physician-patient relationship seemed to be an important component in overcoming patient’s fear and anxiety, that prevented the physician from developing patient HL, particularly in citizens who belong to a different nationality from the country where they reside [27,34-36,41,48].

Barriers

Physicians referred to specific factors that prevent them from developing patients’ HL, such as systemic factors, lack of available time, cultural, socio-economic, language and medical jargon barriers.

Systemic Factors

Physicians argued that there are factors stemming from the dysfunction of the health system, such as lack of staff, large number of patients, work stress, etc. that burden them and prevent them from developing HL for their patients [33,36,37,43,46].

“I wish we had half an hour for every patient, it would be excellent, but in public hospitals, where you have, you know, 12 patients you have to see in 2-3 hours, you can’t really manage to give them enough time, so you always give, you know, points, like dot points of information, and you can’t really explain everything.” (Physician) [33].

“In the ideal world, as I think you’re eluding to, what we’d have are primary care physicians that would be set up in a scenario where they would not be having to see 50 patients a day, but be able to see 25 patients a day.” (Physician) [36].

“When we’re short staffed it puts people under pressure and you tend to have to prioritise things so you tend to do the more urgent stuff and the conversations that really build and support health literacy tend to fall away.” (Physician) [37].

Another factor contributing to the dysfunction of the healthcare system where physicians worked, burdening both the physicians themselves and the patients, was the fragmentation existing between and within services [43].

Time Constraints

Physicians reported the suffocating time conditions under which they work which results in a reduction in their time availability towards their patients [33,34,36,37,39,44,46].

“ (…) the conversations that really build and support health literacy tend to fall away when there is long waiting lists, you know and there are people in the waiting room and it is chaotic.” (Physician) [37].

“I think it’s almost impossible to have a conversation ... with a new patient, [about] your disease, your stage, your prognosis, management, consent, and then goals of care at the same time… we have 15 minutes to see each patient, so I don’t think that’s possible.” (Physician) [44].

The duration of a session between a patient and physician did not always seemed to be sufficient for the physician who wanted to simplify medical jargon and developed his patient skills [30,37].

“You need to do the right thing with the right person. With some people you take the time to look at the information together, to evaluate it together. But there are also situations where you say no, I don’t want to go into it. You have to consider, evaluate and grade, you need to weed some things out and to keep others.” (Physician) [30].

Time spent between physician and patient remained crucial even for those with high HL levels. Their extensive knowledge meant they required more time, often asking complex or more thorough questions [45].

“High literacy patients take a long time because they ask quite complex questions that you wouldn’t expect patients to ask, and very low literacy patients have the paternalistic ‘I don’t want to know’ attitude.” (Physician) [45].

Cultural Barriers

One of the most significant barriers physicians reported encountering in the development of HL was cultural barriers. Different cultures, behaviors, attitudes, customs, traditions, rituals, and more acted as impediments to effective communication between physicians and their patients, hindering the advancement of HL [27,29,33-36,41,44].

Some physicians noted that CALD patients were more influenced by their cultural beliefs than by the physicians themselves [27]. Many times, physicians reported that their beliefs were fatalistic and influenced by cultural norms, leading them to avoid medical examinations. This could have adverse effects on medical treatment and the patients' health.

“For a CALD patient it’s more difficult to explain the treatment because of their cultures and beliefs. They also believe in using traditional medicines and herbal medicines (…).” (Physician) [27].

A notable element was the what a physician noted regarding how these individuals treated him:

“Well, let me put it like this, I’ve always felt that as a doctor, you are … you have the role of a magician. Because many people have a tendency to trust you immediately and completely and that you take care of them and they want to hear they’re doing well, or that it’s not so bad (…).” (Physician) [29].

Socio-Economic Barriers

As revealed through several interviews with physicians in the articles, HL was associated with social class [27,31,33,37,39,40,45-47]. Individuals from lower socioeconomic backgrounds tended to be more passive in their interactions with healthcare professionals and had lower expectations from their physician encounters [31,33,37].

“If the doctor tells them what to do, they go and do it. They generate a huge burden on us because they don’t ask you anything, you see them totally surrendered to what you tell them.” (Physician) [31]

In making a decision about their health treatment, some physicians expressed the view that socioeconomic and socio-environmental factors influenced their treatment choices, always for the benefit of their health [31,39,47].

“It plays a role in the decision, how is the patient’s care situation at home? (…) Is care ensured? And if it is not ensured, in case of an acute event, he has to be admitted to hospital.” (Physician) [39].

“Often people with bad social support have bad transportation issues (and) have difficulties with getting a lot of diagnostic tests done. I think that involving as many people as possible with their care is important.” (Physician) [47].

Physicians indicated that challenging socio-economic conditions are an important barrier to the development of HL [40,46].

“Usually they have a small child, trying to get childcare so you can go to an appointment, to do exercise or have time to cook well. All these things get much more complicated when you have a small child who needs care. So I’d say that is the biggest barrier, that and finances.” (Physician) [40].

“There’s a huge amount of pathology that we don’t know about. There will be lots of silent events and [people’s] health literacy is poor, they struggle with diabetes, education.” (Physician) [46].

Language

Language was cited by some physicians in articles as a barrier to communicating with their patients [27,32-34,36,37,41,45]. Some healthcare providers sometimes conflated HL with general English literacy. When asked how they delivered preventive care to patients with low HL, they often emphasized the importance of addressing language difficulties:

“I think always language has been a barrier.” (Physician) [32].

Some physicians stated that a fundamental prerequisite for discussing treatment with patients was for them to have a good command of the English language.

“There is a gap between patient and GP, the doctor may be unable to understand the patient's problem due to his or her own language barrier. So, the problem can be with the doctor not necessarily with the patient.” (Physician) [27].

“Language barrier: inability to communicate with patients/families and ensure they understand the discussion.” (Physician) [41].

Some physicians had also noted that patients tended to be more open when conversing with them in their native language [33]. Many physicians expressed mistrust and dissatisfaction with translation services [27,33,41].

Medical Jargon

The use of medical jargon could pose a barrier to communication for certain physicians when interacting with patients and, consequently, hinder the development of HL [28,30,41,42,44]. Some physicians explained:

“Doctors and patients ... speak two completely different languages. As if I said “my motor car has 120 horsepower” and one thinks that in my car there are 120 horses riding! The problem is that the patient does not understand the problem of the motorcar; they have a different idea of horses. This is why you need to put things in order (…).” (Physician) [30].

“A lot of our terminology in medicine is almost a separate language really, it’s not part of day-to-day language and we try to put it in a lay person’s language, but the further you go on in medicine, probably the worse you get at doing that as that language to you becomes more day to day.” (Physician) [42].

Some physicians they had state that it was not so easy for them to simplify medical jargon [28,41].

“I noticed that people wanted, needed simpler answers. But I must say, medicine is not that simple and maybe I didn’t know how to simplify to an appropriate level, but people were still asking about the serious health problems of their relatives and this can’t just be simpliﬁed, so this was quite demanding for me.” (Physician) [28].

Discussion

The results of the systematic review reveal that physicians differ in their perception of patients' HL and attribute different meanings to it. Some physicians who attempted to define HL described it as the ability to navigate and utilize the healthcare system, the ability to comprehend prescription instructions and manage medication, or the ability to communicate effectively with their physician.

Although physicians' approaches to managing patients with low HL vary, some prioritize specific communication strategies such as repetition, simplified language, providing written instructions, and more.

Additionally, it appears that HL may influence certain physicians in how they relay medical examination results to their patients or whether they involve patients in treatment decisions, with some showing a preference for engaging patients with high HL in shared decision-making. Trust in the physician-patient relationship seems to be a crucial factor for some physicians in fostering the appropriate conditions for the development of HL.

Many physicians identified various barriers that impede the development of patients' HL. These challenges encompass issues related to the dysfunctional healthcare system, staff shortages, the management of a large patient caseload, limited time, work-related stress, cultural and socio-economic disparities, language barriers, as well as difficulties arising from the use of medical jargon. These findings align with the results of other studies [49-51].

The cultural barriers mentioned by some physicians may arise from inadequate medical education because few academic programs include training for future healthcare providers on managing the health of immigrants [52]. Medical education should address issues stemming from limited HL and cultural differences cohesively, promoting training for culturally competent providers with the ultimate goal of reducing health disparities [53]. Some physicians' observations that HL correlates with socio-economic status appear to align with other studies highlighting the association between low socioeconomic status and low HL [54,55].

No physician mentioned the use of tools to measure patients' HL [56-59], such as the Short Test of Functional Health Literacy in Adults (S-TOFHLA) [56], the Rapid Estimate of Adult Literacy in Medicine (REALM) and the Newest Vital Sign (NVS) [60,61]. These tools are short and quick and easy to use by a physician as they are often used in clinical settings. This omission could potentially lead to an inaccurate assessment of patients' literacy levels, a concern that has been highlighted in studies using quantitative methods to study HL [51,62,63]. The limited knowledge that healthcare professionals possess about HL has been emphasized in previous research [64-66]. Some physicians often overestimate patients' ability to comprehend medical terms or seem to make little effort to adjust the language they use [67,68].

The barrier posed by the use of medical jargon in physician-patient communication seems to be significant, as it has been shown to have a negative impact on patients and their health management [69,70]. In contrast, the use of simplified terms by physicians has been associated with better patient understanding and a greater sense of responsibility and capability to take charge of their healthcare [71]. Additionally, trust in the physician-patient relationship, as mentioned by some physicians, is an essential factor in alleviating patients' fear and anxiety, especially among individuals with low HL, who are more likely to be distrustful of their physicians [72].

Physicians interact with individuals daily, each with varying levels of HL. Through their daily communication with patients, physicians gain valuable experience in understanding and managing HL. Additionally, there is a significant responsibility and role that both the healthcare system and physicians themselves, as healthcare professionals, should play in developing their patients' HL [73,74].

Further research is needed to document physicians' experiences in communicating with patients who have different levels of HL. Simultaneously, medical education in addressing and managing HL should be reinforced through academic curricula and continuing education seminars, among other means. Furthermore, healthcare professionals' working conditions need improvement to ensure that each physician can allocate the necessary time to each patient based on their individual needs, without being hindered by stress-inducing work environments.

Limitations

The number of qualitative and mixed studies that have been conducted regarding physician' perception and understanding of HL are limited [32,33,35,37,42,43,45]. In most of the identified studies, the issue of how physicians handle HL issues is not directly examined or only partially addressed, which in itself constitutes a limitation of the study [27-30,34,36,38,39,41,44,46-48].

The present study chose to extract and analyze only first order constructs in order to produce an interpretive synthesis of the data more secure in terms of its results. Studies not published in the English language or studies that are not accessible have been excluded from this systematic review.

This study underscores the fact that, despite the significant role medical professionals play in the development of their patients' HL, there has been limited research conducted on their perception and understanding of patient HL, as well as the barriers resulting from low HL. This knowledge gap contradicts the actual roles and responsibilities that medical professionals have in contributing to the enhancement of patient HL.

## Conclusions

From the results of the systematic review, it appears that healthcare professionals vary in their perception of patients' HL levels, yet none of them employ a specific measuring tool. Some physicians who sought to define HL diverged in their understanding of its significance. Although there seems to be no uniform approach to managing patients with low HL, some prioritize certain communication strategies. Dysfunctions within the healthcare system, limited available time, cultural and socio-economic barriers, as well as language and medical jargon barriers, appear to hinder physicians who aspire to develop their patients' HL.

Considering that a physician's role is pivotal in the development of patient HL, impacting, among other things, patient-physician communication and treatment effectiveness, there is a need to integrate HL training into medical education. This should occur within a healthcare system that promotes the training of capable healthcare professionals.

The inclusion of patients' HL training is crucial within the academic curricula of universities, both at undergraduate and postgraduate levels, as well as during specialized medical training. Additionally, ongoing educational initiatives-such as seminars and webinars-conducted by state organizations and professional physician associations are essential. These interventions will significantly impact the knowledge, skills, and behaviors of practicing physicians regarding HL.

## Appendices

**Table 3:** Enhancing transparency in reporting the synthesis of qualitative research: ENTREQ Checklist [23]

Item No.	Guide and Description	Report Location
1. Aim	State the research question the synthesis addresses	p. 2
2. Synthesis methodology	Identify the synthesis methodology or theoretical framework which underpins the synthesis, and describe the rationale for choice of methodology (e.g. meta- ethnography, thematic synthesis, critical interpretive synthesis, grounded theory synthesis, realist synthesis, meta-aggregation, meta-study, framework synthesis)	Thematic Analysis p.3
3. Approach to searching	Indicate whether the search was pre-planned (comprehensive search strategies to seek all available studies) or iterative (to seek all available concepts until they theoretical saturation is achieved)	p. 2
4. Inclusion criteria	Specify the inclusion/exclusion criteria (e.g. in terms of population, language, year limits, type of publication, study type)	p. 2
5. Data sources	Describe the information sources used (e.g. electronic databases (MEDLINE, EMBASE, CINAHL, psycINFO), grey literature databases (digital thesis, policy reports), relevant organisational websites, experts, information specialists, generic web searches (Google Scholar) hand searching, reference lists) and when the searches conducted; provide the rationale for using the data sources	p. 2
6. Electronic Search strategy	Describe the literature search (e.g. provide electronic search strategies with population terms, clinical or health topic terms, experiential or social phenomena related terms, filters for qualitative research, and search limits)	p. 2
7. Study screening methods	Describe the process of study screening and sifting (e.g. title, abstract and full text review, number of independent reviewers who screened studies)	pp. 2-3, Fig 1 PRISMA flow diagram
			8. Study characteristics	Present the characteristics of the included studies (e.g. year of publication, country, population, number of participants, data collection, methodology, analysis, research questions)	pp.3-5 Table 1
9. Study selection results	Identify the number of studies screened and provide reasons for study exclusion (e.g. for comprehensive searching, provide numbers of studies screened and reasons for exclusion indicated in a figure/flowchart; for iterative searching describe reasons for study exclusion and inclusion based on modifications to the research question and/or contribution to theory development)	p. 2, Fig 1 – PRISMA flow diagram			
10. Rationale for appraisal	Describe the rationale and approach used to appraise the included studies or selected findings (e.g.	pp. 2-3
